# Curve Sprint Ability as an Indicator of Neuromuscular Function and Physical Fitness in Youth Soccer Players: Comparative Insights from U14 and U16 Groups

**DOI:** 10.3390/medicina61111981

**Published:** 2025-11-05

**Authors:** Zarife Pancar, Burak Karaca, Yung-Sheng Chen, José Antonio Sánchez Fuentes, Santiago Navarro Ledesma, José Carlos Barbero-Álvarez, Francisco Tomás González-Fernández

**Affiliations:** 1 Department of Physical Education and Sports, Faculty of Sports Science, Gaziantep University, Gaziantep 27350, Turkey; 2 Department of Exercise and Health Sciences, University of Taipei, Taipei 111, Taiwan; poko1242@hotmail.com; 3 Exercise and Health Promotion Association, New Taipei City 241, Taiwan; 4 Research Group Movement Sciences and Sport (MS&SPORT), Department of Physical Activity and Sport, Faculty of Sports Sciences, University of Murcia, 30720 San Javier, Spain; 5 Department of Physiotherapy, Faculty of Health Sciences, Campus of Melilla, University of Granada, Querol Street 5, 52004 Melilla, Spain; snl@ugr.es; 6 Department of Physical Education and Sports, Faculty of Education and Sport Sciences, University of Granada, Melilla Campus, 52005 Melilla, Spain; jcba@ugr.es; 7 Department of Physical Education and Sports, Faculty of Sport Sciences, Sport and Health University Research Institute (iMUDS), University of Granada, 18071 Granada, Spain; ftgonzalez@ugr.es

**Keywords:** soccer, aerobic capacity, neuromuscular function, physical fitness, adolescent, agility

## Abstract

*Background and Objectives*: In modern soccer, players frequently perform curved sprinting (CS) actions that combine speed, agility, and neuromuscular control, highlighting the importance of assessing CS performance in youth athletes. This study aimed to investigate age-related differences in CS performance and its relationship with key components of physical fitness—including aerobic capacity, vertical jump height, and agility-based field tests—in U14 and U16 male soccer players. *Materials and Methods*: A total of 76 registered players participated, evenly divided between the two age groups. Statistical analyses included t-tests, correlation, and regression modeling to determine key predictors of curve-sprint performance. *Results*: U16 players showed significant between-group differences, with faster CS times on both the good and weak sides (*p* < 0.001, d = 0.93–1.08), as well as superior performance in the countermovement jump (*p* < 0.001, d = 1.12), Illinois Agility Test (*p* < 0.01, d = 0.70), and 5-0-5 change-of-direction (COD) test (*p* < 0.01, d = 0.74). Significant negative correlations were found between CMJ height and CS times (r = –0.40 to –0.73), indicating that greater explosive power was associated with better CS performance. Moderate to very strong positive correlations were observed between CS and agility measures (r = 0.43 to 0.79), particularly in the U16 group, whereas VO_2_max showed no meaningful relationship with CS performance (r = –0.16 to –0.30, *p* > 0.05). *Conclusions*: These results suggest that CS may serve as an indicator of neuromuscular power and agility, both of which improve with age. From a practical perspective, regular curve-sprint assessments and multidirectional drills may support talent development by helping coaches monitor neuromuscular efficiency and movement coordination in young players.

## 1. Introduction

Soccer is a sport characterized by its high physical and physiological demands, requiring athletes to execute a broad range of high-intensity, multidirectional actions such as sprinting, jumping, and rapid changes in direction [1,2,3]. These explosive efforts are critical for match performance and frequently occur during key phases of play, including counterattacks, transitions, and defensive recoveries [4,5]. In youth academy and regional competitions, players often perform high-intensity actions requiring a combination of speed, agility, and neuromuscular control, highlighting the importance of physical conditioning in youth football [6,7]. Among these actions, sprinting is one of the most frequent and decisive movements influencing performance. It can be broadly categorized into linear sprinting (LS) and curve sprinting (CS). Although LS has been extensively studied, researchers have recently focused more on CS due to its prevalence in real match situations [8]. Video-based match analyses have shown that up to 86% of sprinting actions in soccer follow curved trajectories, rather than straight lines [9]. This curvilinear sprinting pattern requires specific biomechanical and neuromuscular adaptations, including asymmetrical force production, dynamic balance, trunk control, and the ability to maintain velocity while turning [10,11]. These demands highlight the need to assess and train CS independently of LS to improve soccer-specific sprint performance.

Although previous studies have reported moderate to strong correlations between CS and other physical qualities such as LS, COD, and jumping ability, these attributes are not entirely interchangeable [9,12]. For example, proficiency in LS does not necessarily predict CS performance, as the latter involves different neuromechanical and coordinative demands [13]. Therefore, it is necessary to adopt a multidimensional training approach that addresses each of these physical attributes independently. Age is another critical factor influencing athletic performance. As players progress through adolescence, their physical capacities improve due to biological maturation, hormonal changes, and cumulative training exposure [14,15]. These developmental processes lead to improvements in neuromuscular coordination, eccentric strength, and movement efficiency, which may contribute to the performance differences observed between younger and older youth players [16,17]. The Yo-Yo IR1 test is widely recognized for its ability to assess intermittent endurance, a measure strongly linked to overall match performance and repeated sprint ability, including CS execution [18,19]. Similarly, field-based agility tests such as the IAT and V-Cut provide insight into an athlete’s capacity to perform rapid and efficient directional changes, a fundamental aspect of curvilinear sprinting [20,21]. Recent evidence indicates that agility and power-based tests are significant predictors of CS performance, with correlation coefficients ranging from r = 0.50 to 0.78, depending on the athlete’s age and the specific test used [21,22,23]. These findings support the inclusion of curve–sprint-specific drills and multidirectional movement training within youth development programs.

Given the limited research exploring CS in youth athletes, particularly across age categories, this study aimed to examine age-related differences in CS performance and its associations with key physical fitness variables, namely, aerobic capacity, lower limb explosive power, and agility/change of direction abilities in U14 and U16 soccer players [21]. However, there was a lack of comparative research examining how the relationship between curve sprint performance and key physical fitness parameters (such as jump ability, agility, and aerobic capacity) differs across distinct adolescent age groups, particularly between U14 and U16 soccer players. By identifying age-related differences and key predictors of CS ability, this study aimed to provide evidence-based recommendations for improving multidirectional speed, explosive strength, and movement efficiency, attributes that are essential for high-level performance in contemporary soccer. Curve sprinting inherently represents the neuromuscular and biomechanical efficiency of the athlete, as it requires asymmetric force generation, inter-limb coordination, and rapid eccentric–concentric transitions. These mechanisms are fundamental determinants of movement control and power transmission during high-speed, multidirectional actions in soccer. Therefore, beyond its correlations, curve sprint performance can be regarded as a functional indicator of neuromuscular function and agility capacity. This rationale justifies the present study’s aim to examine whether differences in curve sprint ability between age categories reflect distinct stages of neuromuscular maturation and physical fitness development in youth soccer players. Therefore, it was hypothesized that (1) U16 players would show superior curve-sprint and physical fitness performance compared to U14 players due to greater neuromuscular maturation and training experience, and (2) curve sprint performance would be positively associated with explosive power and agility measures, reflecting its role as an indicator of neuromuscular function in youth soccer.

## 2. Materials and Methods

### 2.1. Experimental Approach to the Study

**Figure 1:**
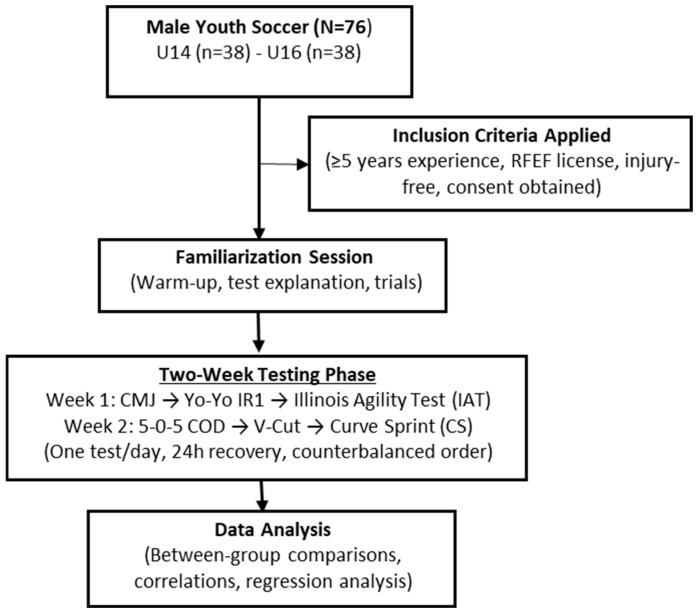
Experimental Flow Diagram.

This study employed a cross-sectional, comparative, and correlational design to examine age-related differences in curve sprint (CS) performance and its associations with aerobic capacity, jump performance, and change of direction (COD) agility among youth male soccer players (U14 and U16). Data collection was carried out in March 2025 at the training facilities of a professional soccer academy in Granada, Spain. All assessments were performed on synthetic turf, following standardized testing protocols and under controlled environmental conditions. During testing sessions, the ambient temperature ranged between 15 °C and 18 °C, and the relative humidity ranged from 50% to 60%. Testing days were scheduled to avoid adverse weather conditions such as rain or strong winds. All assessments were conducted over two consecutive weeks, with one test per day and a minimum of 24 h of recovery between sessions to minimize the effects of fatigue. Participants were recruited from the same academy and tested at similar times of the day to reduce circadian rhythm effects. Before each session, players completed a standardized dynamic warm-up, and adequate recovery periods were provided between trials. To control for metabolic and neuromuscular fatigue, the physical tests were distributed over two weeks, ensuring no overlap between major testing days. Prior to participation, players and their legal guardians received detailed verbal and written information regarding the purpose, procedures, potential risks, and benefits of the study and provided written informed consent. This study was conducted in accordance with the Declaration of Helsinki and received ethical approval from the University of Granada Research Ethics Committee (approval code: 4712/CEIH/2024, issued on 16 December 2024). The following neuromuscular and performance tests were conducted under standardized conditions: Countermovement Jump (CMJ), Curve Sprint (CS), V-Cut Agility, Illinois Agility Test (IAT), 5-0-5 Change of Direction (COD), and Yo-Yo Intermittent Recovery Level 1 (Yo-Yo IR1). The overall experimental design and testing sequence are illustrated in Figure 1.

### 2.2. Participants

A total of seventy-six male youth soccer players participated voluntarily in this study, comprising two age categories: U14 (n = 38) and U16 (n = 38). The U14 group (n = 38; age: 13.8 ± 0.4 years, height: 161.5 ± 5.9 cm, body mass: 52.4 ± 6.3 kg) and the U16 group (n = 38; age: 15.7 ± 0.5 years, height: 172.8 ± 6.7 cm, body mass: 63.8 ± 7.0 kg) participated in the study. All participants were healthy, injury-free, and engaged in regular training (five sessions per week) within the same professional academy in Granada, Spain.

The inclusion criteria were as follows: official registration under the Royal Spanish Football Federation (RFEF); a minimum of five years of structured soccer experience; and signed informed consent from both players and their legal guardians. A priori sample size calculation was conducted using G*Power (version 3.1; Heinrich Heine University, Düsseldorf, Germany; accessed on 15 March 2025). The analysis assumed a two-tailed test with α = 0.05 and power (1 – β) = 0.95, considering a medium effect size (d ≈ 0.60 for group comparisons, r ≈ 0.35 for correlations). Results indicated that a minimum of 60 participants would be required to achieve adequate statistical power. The present sample (n = 76) therefore exceeded this requirement, ensuring sufficient power for both between-group and correlational analyses [24]. Although biological maturation was not directly measured, players were recruited from well-defined competitive age categories (U14 and U16), which typically correspond to chronological and developmental stages in youth soccer. Future studies are encouraged to include maturation indicators such as peak height velocity (PHV) or Tanner staging to better account for inter-individual variability.

### 2.3. Test Procedures

All testing sessions were supervised by certified strength and conditioning coaches and sport scientists. Prior to testing, participants completed a standardized warm-up consisting of 5 min of light jogging, dynamic stretching (e.g., hip openers, leg swings, lunges), and multidirectional drills involving accelerations, decelerations, and COD movements. The order of testing was counterbalanced across participants to minimize fatigue and learning effects. All assessments were conducted under standardized laboratory and field conditions, with one test performed per day to prevent fatigue. Each test included two maximal trials, with rest intervals adjusted according to the specific test protocol. During recovery periods, participants remained seated or standing quietly to ensure consistency across sessions.

Testing Order and Recovery. All tests were administered over a two-week period in the following sequence to minimize fatigue: Countermovement Jump (CMJ), Yo-Yo Intermittent Recovery Test Level 1, Illinois Agility Test (IAT), 5-0-5 Change of Direction (COD) Test, V-Cut Test, and Curve Sprint (CS) Test (performed in both directions). The testing order was intentionally structured to progress from neuromuscular to agility and finally endurance assessments, in alignment with previous field-based studies [25]. Each participant performed two to three trials per test, and the best score was retained for analysis. Passive rest intervals of 1–3 min between trials and 10–15 min between tests were provided to ensure full recovery. Prior to data collection, all participants were familiarized with the testing procedures. Each test was explained and demonstrated using standardized equipment to ensure proper understanding and execution.

Countermovement Jump Test. The CMJ was used to evaluate explosive lower-limb power and vertical jump performance. This test is widely used in football and other team sports as a reliable indicator of neuromuscular function, particularly for actions involving sprinting, jumping, and change of direction [26]. Measurements were obtained using a Chronojump Boscosystem^®^ contact platform (Chronojump, Barcelona, Spain) [27]. Participants began from an upright standing position with hands placed on the hips, performed a rapid downward movement (eccentric phase), and immediately jumped vertically (concentric phase). Arm swing was restricted to isolate lower-limb power output. Each player performed three maximal efforts, with 30–45 s of passive rest between trials. The highest jump height (cm) was retained for analysis. The CMJ test has demonstrated excellent test–retest reliability in both youth and elite athletes (ICC > 0.90).

Yo-Yo Intermittent Recovery Test Level 1 (Yo-Yo IR1). The Yo-Yo IR1 was used to estimate players’ aerobic endurance capacity. This field-based test closely simulates the intermittent nature of football and is widely recognized as a valid and practical method for evaluating fitness in youth soccer players [28]. The test consists of 20-m shuttle runs performed at progressively increasing speeds, guided by audio beeps. Each shuttle is followed by 10 s of active recovery (walking). The test continues until the participant fails to reach the 20 m line on two consecutive occasions or voluntarily stops due to fatigue. Although VO_2_max is less directly related to short anaerobic actions such as curved sprints, it was included to assess the potential contribution of aerobic capacity to recovery and repeated-sprint performance, which are essential to the demands of youth football matches. The total distance covered (in meters) is recorded. Estimated VO_2_max was calculated using the formula proposed by Bangsbo et al. [18]: VO_2_max (mL·kg^−1^·min^−1^) = (IR1 distance in m × 0.0084) + 36.4.

Illinois Agility Test (IAT). The test was employed to evaluate players’ ability to rapidly change direction and navigate a slalom-style course. The test was conducted on a 10 × 5 m layout using four central cones spaced 3.3 m apart and four additional cones marking the start, finish, and turning points. Players began each trial in a prone position with their hands at shoulder level. Timing was measured using electronic photocell gates (Microgate Wireless Training Timer, Bolzano, Italy). Typical error of the photocells was between 0.04 and 0.06 s, while the smallest worthwhile change was between 0.11 and 0.17 s [29]. Each participant completed two attempts, with the fastest time used for statistical analysis. This test has demonstrated high validity and reliability in assessing agility among youth athletes [30,31,32].

#### 5-0-5 Change of Direction (COD) Test

The 5-0-5 test was employed to evaluate the athletes’ short-distance change of direction ability, including deceleration, 180° turning, and re-acceleration. Each participant began from a standing start, sprinted 10 m, executed a sharp 180° turn on a designated line using either the dominant or non-dominant leg, and then sprinted back 5 m. Timing was measured in seconds using electronic photocell gates placed at the 5 m return line. Two trials were performed for each leg, with a 2-min rest interval between attempts. The fastest time recorded across the trials was used for statistical analysis. This test has been widely validated for assessing agility and COD performance in youth soccer players [33,34].

V-Cut Agility Test. The test was used to evaluate players’ multidirectional speed and change-of-direction ability. The test was performed on a 25 m course involving four sharp directional changes, forming a V-shaped running pattern. Timing was measured using photocell gates to ensure accuracy. Each player completed two trials, with 2 min of passive rest between attempts. The fastest completion time (in seconds) was recorded for statistical analysis. Due to its similarity to game-like movement patterns, the V-Cut test is considered a valid field-based measure of agility in football-specific populations [35,36].

Curve Sprint (CS) Test. The test was conducted to evaluate players’ ability to accelerate and maintain maximal speed along a curved trajectory, a movement highly specific to football. The protocol followed standardized procedures reported in previous studies [37], which have demonstrated high validity and reliability for evaluating curvilinear sprint performance in youth soccer players. In this study, players sprinted along a semi-circular path with a radius of 9.15 m and an arc distance of 17 m, consistent with official curve sprint protocols. Timing gates were positioned at the start (0 m) and finish (17 m) lines. Participants began from a split-stance position, with the front foot approximately 1 m behind the first gate to prevent early triggering. Each participant performed three trials to the right and three to the left, with 3 min of passive recovery between attempts. The best time on each side was selected for analysis. The side on which the player achieved the fastest time was categorized as the ‘good side’ (GS), and the slower side as the ‘weak side’ (WS), in line with previous studies [37]. The CS test has shown excellent test–retest reliability (ICC > 0.90) and sensitivity for distinguishing between age groups and performance levels in football players.

### 2.4. Statistical Analyses

The statistical analysis of the data obtained in the study was performed using SPSS statistical software (SPSS for Windows, version 25.0, IBM Corp., Armonk, NY, USA). All analyses were conducted as two-tailed with α = 0.05. Descriptive statistics are reported as mean ± SD. Distributional assumptions were first screened using the Shapiro–Wilk test and variance homogeneity with Levene’s test. In addition, Skewness and kurtosis values within ±2 were considered acceptable for normality at the group level [38]. Between-group (U14 vs. U16) comparisons of physical performance variables were tested with independent-samples Student’s t-tests; effect sizes were expressed as Cohen’s d and interpreted as small (0.2), medium (0.5), and large (≥0.8) [39]. The primary inferential approach evaluated whether curve sprint time (CS, s) is indicated by neuromuscular and agility measures while accounting for repeated measures (Good vs. Weak side) within players. We fitted linear mixed-effects models of the form: CS (s) ∼ Predictor + Age Group + Side + Predictor × Age Group + (1 Player ID), where Predictor was entered one at a time (CMJ [cm], Illinois [s], 5-0-5 [s], V-Cut [s], VO_2_max [mL·kg^−1^·min^−1^]; age group (U16 = 1, U14 = 0) and Side (Weak = 1, Good = 0) were fixed factors; and a random intercept for Player ID modeled the within-player dependence of Good/Weak trials. Parameters were estimated by REML; Wald 95% confidence intervals and two-tailed *p*-values are reported. For interpretability, unstandardized slopes (β) are expressed in seconds per unit of the predictor (e.g., s·cm^−1^ for CMJ). We additionally report marginal R^2^ (variance explained by fixed effects) following Nakagawa and Schielzeth. To aid practical interpretation, we present expected changes in CS for fixed increments (+5 cm CMJ; +1.0 s for agility tests; +5 mL × kg^−1^ × min^−1^ VO_2_max): ΔCS = β × ΔX. Pairwise Pearson correlations between CS and the fitness measures were computed as a secondary exploratory analysis. When multiple within-family tests were performed, the Benjamini–Hochberg false discovery rate (FDR) correction was applied, and q-values are reported (*) when q < 0.05.

## 3. Results

**Figure 2:**
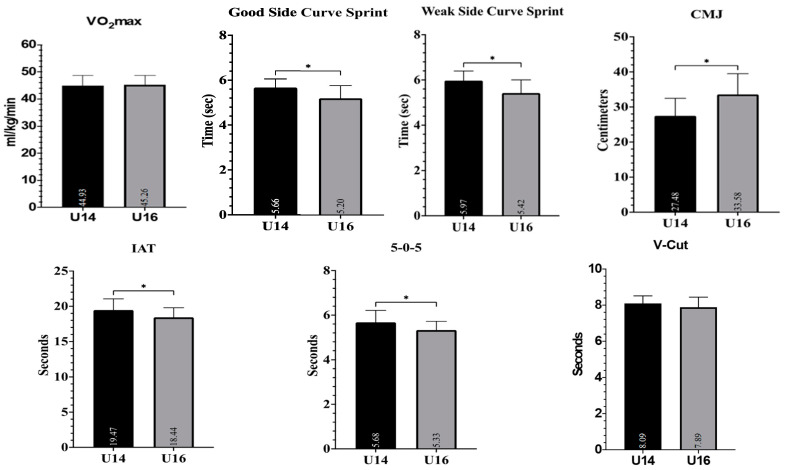
Group comparison (U14 vs. U16) for physical performance tests: VO_2_max (Yo-Yo IR1; mL·kg^−1^·min^−1^), curve sprint test (CS; seconds), countermovement jump (CMJ; cm), Illinois Agility Test (IAT; seconds), 5-0-5 Change of Direction Test (seconds), and V-Cut test (seconds). Bars represent mean ± SD values for each group. * indicates a significant difference between groups (*p* < 0.05).

**Figure 3:**
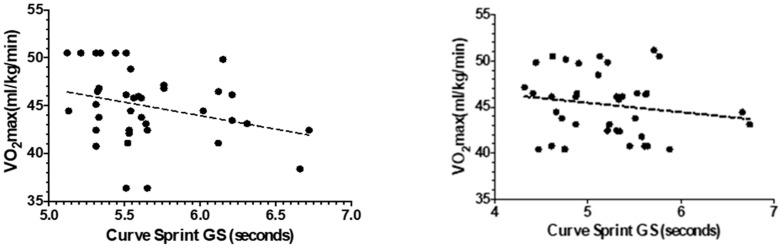
Correlation between VO_2_max and Curve Sprint Performance (Good Side) in U14 (**left graph**) and U16 (**right graph**) Soccer Players.

**Figure 4:**
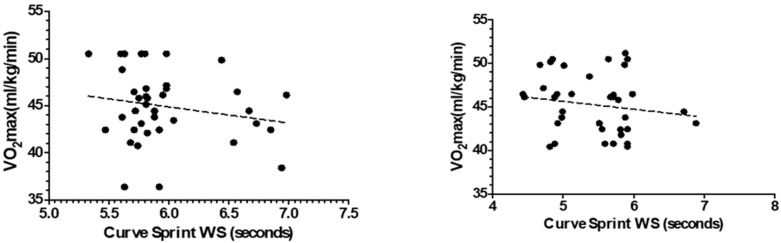
Correlation between VO_2_max and Curve Sprint Performance (Weak Side) in U14 (**left graph**) and U16 (**right graph**) Soccer Players.

**Figure 5:**
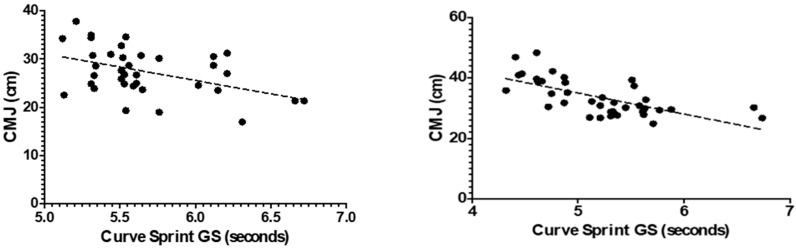
Correlation between Countermovement Jump (CMJ) and Curve Sprint Performance (Good Side) in U14 (**left graph**) and U16 (**right graph**) Soccer Players.

**Figure 6:**
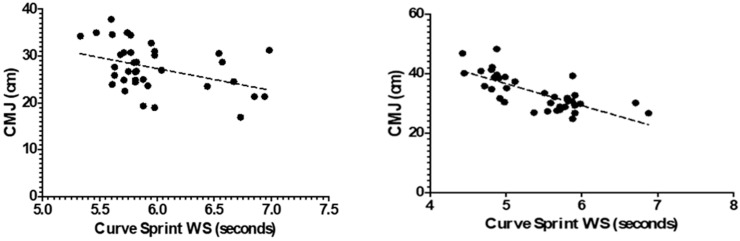
Correlation Between Countermovement Jump (CMJ) and Curve Sprint Performance (Weak Side) in U14 (**left graph**) and U16 (**right graph**) Soccer Players.

**Figure 7:**
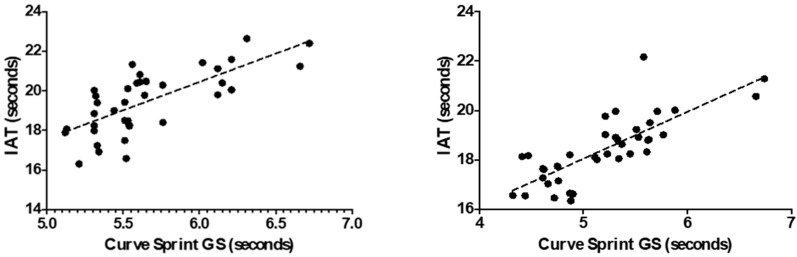
Correlation between Illinois Agility Test (IAT) and Curve Sprint Performance (Good Side) in U14 (**left graph**) and U16 (**right graph**) Soccer Players.

**Figure 8:**
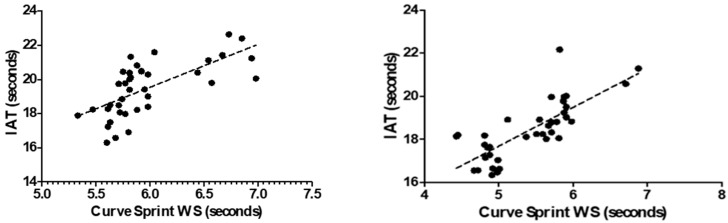
Correlation between Illinois Agility Test (IAT) and Curve Sprint Performance (Weak Side) in U14 (**left graph**) and U16 (**right graph**) Soccer Players.

**Figure 9:**
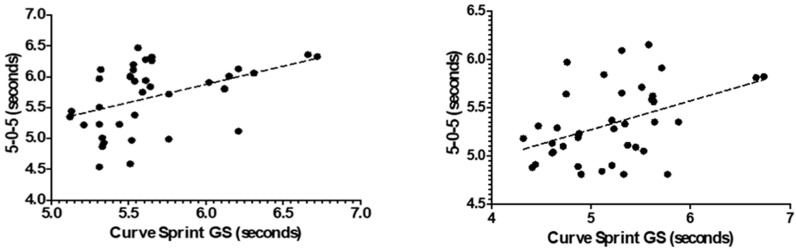
Correlation between 5-0-5 Change of Direction Test and Curve Sprint Performance (Good Side) in U14 (**left graph**) and U16 (**right graph**) Soccer Players.

**Figure 10:**
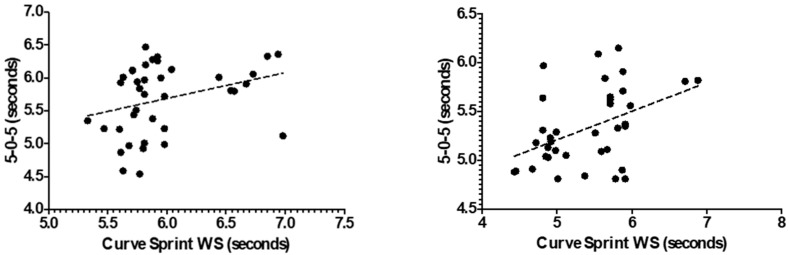
Correlation between 5-0-5 Change of Direction Test and Curve Sprint Performance (Weak Side) in U14 (**left graph**) and U16 (**right graph**) Soccer Players.

**Figure 11:**
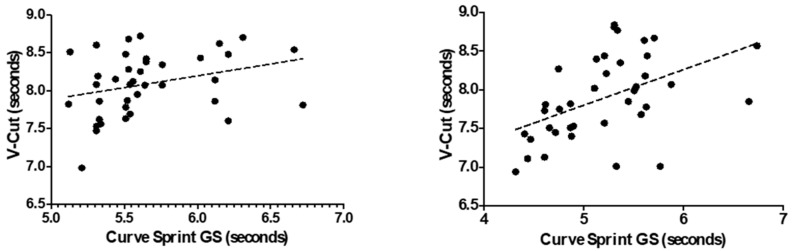
Correlation between V-Cut Test and Curve Sprint Performance (Good Side) in U14 (**left graph**) and U16 (**right graph**) Soccer Players.

**Figure 12:**
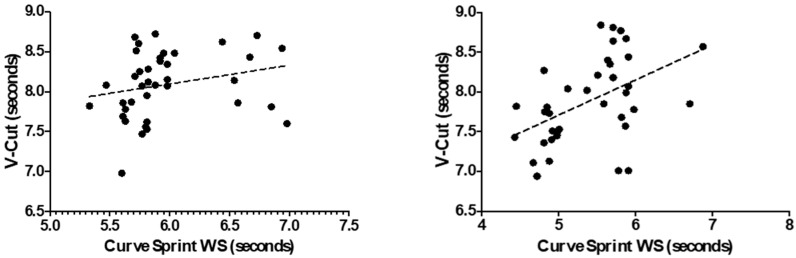
Correlation between V-Cut Test and Curve Sprint Performance (Weak Side) in U14 (**left graph**) and U16 (**right graph**) Soccer Players.

The results are presented in two sections. First, independent-sample t-tests were conducted to compare physical performance variables between the U14 and U16 groups. Second, Pearson correlation analyses were then performed separately for each group to evaluate the relationships between CS performance and aerobic capacity, jump height, and agility-based test outcomes. Detailed descriptive statistics and significant findings are summarized in Table 1 and Table 2 and Figure 2, Figure 3, Figure 4, Figure 5, Figure 6, Figure 7, Figure 8, Figure 9, Figure 10, Figure 11 and Figure 12.

Table 1 presents the comparison of physical performance variables between U14 and U16 soccer players. The U16 group demonstrated significantly faster times in the curve sprint on both the good (5.20 ± 0.56 s) and weak sides (5.42 ± 0.59 s) compared to the U14 group (5.66 ± 0.40 s and 5.97 ± 0.42 s, respectively; *p* < 0.001 for both, d = 0.93 and d = 1.08). Similarly, the U16 players achieved significantly greater jump heights in the countermovement jump (33.58 ± 5.90 cm) than U14 players (27.47 ± 4.99; *p* < 0.001, d = −1.117). Agility tests also favored the U16 group, showing significantly better results in the Illinois Agility Test (*p* = 0.003) and 5-0-5 test (*p* = 0.002). However, no significant difference was observed in VO_2_max values between the two groups (*p* = 0.687, d = −0.09). The V-Cut test approached statistical significance (*p* = 0.081), with a moderate effect size (d = 0.406), indicating a trend towards better performance in U16 players. Visual representations of these group differences are provided in Figure 1.

Table 2 presents the correlations between curve sprint performance and VO_2_max, countermovement jump (CMJ), and agility tests (IAT, 5-0-5, V-Cut) in U14 soccer players. Curve Sprint GS (good side) time showed a strong positive correlation with the Illinois Agility Test (r = 0.719, *p* < 0.001), indicating that slower curve sprint times are associated with poorer agility performance. A significant positive correlation was also found with the 5-0-5 test (r = 0.438, *p* = 0.006). CMJ was significantly and negatively correlated with GS sprint time (r = −0.445, *p* < 0.001), suggesting that players with better jump performance tend to have faster curve sprint times. Correlations with VO_2_max and the V-Cut test approached but did not reach statistical significance (*p* > 0.05). Similarly, WS (weak side) curve sprint time was strongly positively correlated with IAT (r = 0.669, *p* < 0.001) and negatively correlated with CMJ (r = −0.399, *p* = 0.013). The correlation with the 5-0-5 test approached significance (r = 0.311, *p* = 0.058), while the relationships with VO_2_max and V-Cut were not statistically significant (*p* > 0.05).

Table 3 presents the correlations between curve sprint times and VO_2_max, CMJ, and agility tests (IAT, 5-0-5, V-Cut) in U16 soccer players. Curve Sprint GS (good side) time showed a very strong positive correlation with the Illinois Agility Test (r = 0.785, *p* < 0.001), indicating that slower curve sprint times are associated with poorer agility performance. Significant positive correlations were also observed with the V-Cut (r = 0.477, *p* = 0.002) and 5-0-5 tests (r = 0.430, *p* = 0.007). CMJ was strongly and negatively correlated with GS sprint time (r = −0.661, *p* < 0.001), suggesting that better jump performance is linked to faster curve sprint performance. The correlation with VO_2_max was not statistically significant (*p* = 0.319). Similarly, WS (weak side) curve sprint time was strongly positively correlated with IAT (r = 0.775, *p* < 0.001) and significantly negatively correlated with CMJ (r = −0.729, *p* < 0.001). Positive and statistically significant correlations were also observed with the 5-0-5 test (r = 0.442, *p* = 0.005) and V-Cut test (r = 0.478, *p* = 0.002). The relationship with VO_2_max was again not statistically significant (*p* = 0.351). Table 4 presents the percentage values of curve sprint directional asymmetry between the good and weak sides.

Mixed-effects regression (random intercept for player) indicated that curve-sprint time (CS) is predicted by neuromuscular and agility-based tests rather than aerobic capacity (Table 5). Specifically, CMJ showed a negative slope in both age groups (U14: β = −0.035 s·cm^−1^, 95% CI = −0.060 to −0.010, *p* = 0.006; U16: β = −0.068 s·cm^−1^, 95% CI = −0.089 to −0.047, *p* < 0.001), with a steeper effect in U16 (interaction: *p* = 0.048, R^2^ = 0.522). The Illinois test displayed a positive association with CS (U14: β = +0.179 s·s^−1^, 95% CI = 0.115–0.244, *p* < 0.001; U16: β = +0.329 s·s^−1^, 95% CI = 0.254–0.405, *p* < 0.001), again stronger in U16 (interaction: *p* = 0.003, R^2^ = 0.662). The 5-0-5 and V-Cut tests showed smaller, age-amplified effects (U16: β = +0.643 and +0.506 s·s^−1^, both *p* < 0.001; R^2^ ≈ 0.37), whereas VO_2_max was not a reliable predictor (U14 *p* = 0.211; U16 *p* = 0.248; R^2^ = 0.267). Practically, a +5 cm increase in CMJ corresponds to approximately −0.17 s (U14) and −0.34 s (U16) faster CS times, whereas a +1.0 s increase in Illinois performance corresponds to approximately +0.18 s (U14) and +0.33 s (U16) slower CS (see ‘Practical ΔX→ΔCS’).

Figure 3 and Figure 4 illustrate the relationship between VO_2_max and curve sprint performance for both good and weak sides, showing weak and statistically non-significant correlations in both U14 and U16 groups. Figure 5 and Figure 6 highlight significant negative associations between CMJ and curve sprint times, indicating that players with higher jump performance completed the curve sprint in shorter times. Strong positive correlations are evident in Figure 7 and Figure 8 between IAT results and sprint performance, suggesting that slower sprint times are associated with poorer agility. Similarly, Figure 9 and Figure 10 show moderate positive correlations between 5-0-5 change-of-direction performance and curve sprint times. Finally, Figure 11 and Figure 12 show that V-Cut test times were positively correlated with curve sprint performance, with stronger associations found in the U16 group. Overall, U16 players consistently demonstrated superior neuromuscular and agility-related performance compared with U14 players, confirming the expected age-related progression in curve sprint ability and overall physical fitness.

## 4. Discussion

This study aimed to examine the relationships between CS performance and various physical fitness parameters, including aerobic capacity (VO_2_max), neuromuscular power (CMJ), and agility/change of direction (IAT, 5-0-5, and V-Cut tests) in young male soccer players categorized into U14 and U16 age groups. The findings provide novel insights into how these physical attributes interact with curved sprinting ability, a key component in modern soccer performance.

In the U14 group, CMJ showed a moderate negative correlation with CS performance, indicating that players with greater vertical jump ability tended to achieve faster curve sprint times. This observation is consistent with previous findings suggesting that neuromuscular power—particularly lower-limb explosive strength—contributes to more effective force application during sprinting, especially along non-linear trajectories [37,40]. CMJ performance reflects rapid recruitment and synchronization of motor units, which are also essential during the ground contact phases of curved sprints where eccentric–concentric muscle actions must occur efficiently [41]. In addition to correlation analyses, the mixed-effects regression results further confirmed that neuromuscular and agility-based variables, particularly CMJ and Illinois test performance, significantly predicted curve sprint time, while aerobic capacity (VO_2_max) did not emerge as a reliable predictor. This reinforces the notion that non-linear sprint ability depends primarily on explosive strength and change-of-direction skills rather than aerobic endurance. The steeper regression slopes observed in the U16 group suggest maturational and training-related improvements in the predictive contribution of neuromuscular qualities to curve sprint performance. The biomechanical demands of curve sprinting further highlight the importance of neuromuscular coordination: unlike linear sprinting, curvilinear movement requires athletes to generate asymmetric forces between the inner and outer legs while maintaining balance, trunk stability, and horizontal velocity [9,11,42]. Similarly, agility-based tests such as the IAT, 5-0-5, and V-Cut demonstrated significant positive associations with CS performance, as they assess an athlete’s ability to decelerate, reorient, and reaccelerate efficiently—movement patterns that are biomechanically similar to curve sprinting [43,44]. Slower agility times were linked to poorer CS outcomes, likely reflecting lower reactive strength, eccentric braking ability, and inter-limb coordination—key components of multidirectional performance. Physiologically, these results may relate to the predominant anaerobic energy system contribution during short, high-intensity efforts such as curve sprinting. In younger athletes, anaerobic enzyme activity, phosphocreatine availability, and type II muscle fiber recruitment are still developing [15]. Therefore, players with more advanced neuromuscular profiles—whether due to early biological maturation or greater training exposure—may possess a potential advantage in tasks requiring explosive power and rapid directional control. Collectively, these findings emphasize the multifactorial nature of curve sprint performance and suggest the importance of training approaches that develop both linear and non-linear sprint mechanics, with particular attention to eccentric strength, coordination, and neuromuscular reactivity during early athletic development. Consistent with this developmental progression, stronger relationships were observed in the U16 group. CMJ displayed a strong negative correlation with CS times, indicating that enhanced muscular power tends to accompany better curve sprint performance. Similarly, the IAT, 5-0-5, and V-Cut tests showed strong positive associations with CS performance, supporting the contribution of agility and change-of-direction (COD) ability to curvilinear sprinting efficiency. These results align with Negra et al. [11], who reported age-related improvements in neuromuscular coordination and strength during adolescence.

Interestingly, VO_2_max did not show a significant correlation with CS performance in either the U14 or U16 cohorts, suggesting that aerobic capacity may play a limited role in short, high-intensity, and predominantly anaerobic tasks. This finding aligns with previous evidence indicating that CS performance relies more on neuromuscular and anaerobic mechanisms—such as phosphocreatine availability, reactive strength, and coordination—than on aerobic metabolism [15,18]. While aerobic fitness contributes to recovery and repeated-sprint ability, the metabolic specificity of CS (a sub-7-s effort) likely reduces its dependence on oxygen transport and utilization systems. Therefore, improvements in VO_2_max with age may not directly translate into enhanced curvilinear sprint performance. These findings are consistent with Faude et al. [45], who reported that short-duration sprints and directional changes are primarily linked to neuromuscular power rather than aerobic endurance, especially in efforts lasting under 10 s. Aerobic fitness appears more relevant for recovery between repeated high-intensity bouts (e.g., Yo-Yo IR1) than for isolated tasks such as CS. Therefore, while VO_2_max remains an important component of overall soccer performance, its direct association with curvilinear sprint ability seems limited when assessed outside of game-specific or repeated-sprint contexts. These results highlight the importance of test specificity in evaluating physical qualities in youth athletes and suggest that coaches should emphasize neuromuscular and anaerobic development—through plyometric exercises, resisted sprints, and curve-specific drills—to enhance CS performance. Endurance training aimed at improving VO_2_max may be best implemented separately from CS-oriented sessions.

An interesting aspect of this study is the age-related influence on physical performance outcomes. Although both U14 and U16 groups displayed similar correlation patterns between CS and the other performance variables, the U16 players exhibited stronger and more consistent associations with agility and power measures, such as CMJ, IAT, 5-0-5, and V-Cut. This trend likely reflects maturation-related improvements in neuromuscular coordination, eccentric strength, and inter-limb efficiency [15,46]. During adolescence, motor unit recruitment, neural firing rate, and muscle fiber hypertrophy improve substantially under hormonal influences such as increased testosterone and growth hormone [47,48]. These biological adaptations contribute to enhanced movement precision, postural control, and force production—all essential for performing complex motor tasks like curve sprinting. Moreover, greater exposure to structured training with age may support technical and biomechanical refinements, including foot placement, trunk rotation, and braking strategies, which are crucial for agility and efficient change of direction [49]. Cognitive and perceptual development may also play a role, as older players tend to demonstrate better anticipation and decision-making skills, indirectly influencing agility-related test performance [50]. In summary, these findings suggest that age-related biological and motor maturation may strengthen the associations between physical attributes and curved sprint performance. This highlights the potential value of age-specific training strategies that emphasize motor learning and neuromuscular development in younger athletes while progressively integrating more intensive and task-specific strength and agility training in older groups.

The present findings are consistent with previous biomechanical investigations that have highlighted the distinctive demands of curve sprinting, including asymmetrical force production, variable stride patterns, and differential limb loading [13,51,52]. Unlike linear sprinting, curve sprinting requires greater concentric force from the outer leg, along with precise braking and redirection ability from the inner leg, to maintain optimal trajectory and velocity. These demands appear to be more efficiently managed by athletes with superior agility, neuromuscular coordination, and power output, as demonstrated by the strong associations observed in the present study, particularly among U16 players. From an applied perspective, these results strongly support the integration of multi-directional plyometric drills (e.g., lateral bounds, single-leg hops) and curve-specific COD drills into youth soccer conditioning programs. Given that nearly 85% of sprint actions during competitive soccer matches involve curved trajectories [9], exclusive focus on linear sprinting in training may be insufficient to meet the real match demands. Incorporating targeted exercises that replicate the mechanical and neuromuscular characteristics of curve sprinting such as resisted curve runs, arc-based agility courses, and asymmetrical strength work can help improve performance in both competitive sprint actions and overall match performance. Moreover, tailoring such programs based on age-related developmental differences, as demonstrated by the differential patterns between U14 and U16 players, will maximize training efficacy and player development over time. This study has several limitations. First, the inclusion of only male players limits the generalizability of the findings across genders. The focus on only two age categories (U14 and U16) restricts the ability to assess broader developmental trajectories across youth soccer. Additionally, the physical tests employed did not incorporate cognitive or technical skill measures, which may also influence curve sprint performance. Finally, the cross-sectional design of the study does not allow for longitudinal assessment of training effects or performance progression over time.

These findings are supported by biomechanical analyses of curved sprinting, which show that sprinting along a curved path imposes distinct mechanical demands compared to linear sprinting. Curved sprints require asymmetrical force production, inter-limb coordination, and precise neuromuscular control due to the centrifugal forces acting on the body [53,54,55,56,57]. Fitzpatrick et al. [46] also noted that players frequently perform curved sprints in match situations, demanding both physical and perceptual adaptations. Efficient management of ankle moments and ground reaction forces appears particularly critical for curvilinear movements [53]. The asymmetrical loading between the inner and outer legs during CS actions may partly explain the observed association between lower-limb explosive strength (CMJ) and CS performance. Yanci et al. [58] similarly found strong relationships between vertical and horizontal jump tests and multidirectional performance, underscoring the relevance of muscular power in acceleration, deceleration, and direction-change tasks. From a developmental perspective, these relationships were more evident in U16 players, who may benefit more from curve-specific sprint training due to greater neuromuscular maturity and strength capacity. These observations align with recent recommendations to integrate curve sprinting within agility and power training programs for youth athletes [54,55]. Furthermore, by including assessments that reflect football-specific movement patterns, such as the V-Cut and IAT, this study extends our understanding of how physical attributes translate into sport-specific performance. In sum, our findings, together with existing literature, reinforce the necessity of age-specific and multidirectional training models that reflect the biomechanical and physiological realities of football, moving beyond the traditional focus on linear sprinting to include curved and reactive sprint components. In addition, recent advances in motion and gait analysis have enabled more detailed evaluations of running mechanics through machine learning and sensor-based systems [59]. Although such approaches differ from traditional field-based testing, they provide a broader framework for understanding neuromuscular coordination and asymmetry in sports performance contexts.

This study has several limitations that should be acknowledged. First, biological maturation was not directly assessed (e.g., peak height velocity or Tanner stage), which may have influenced inter-individual differences in performance, particularly across age groups. Second, participants were recruited from a single soccer academy, limiting the generalizability of the findings to other competitive levels or training environments. Finally, only male players were included in the sample; therefore, the results cannot be generalized to female athletes, who may exhibit different developmental and biomechanical profiles. Future research should address these limitations by including maturation assessments, more diverse training populations, and both sexes to enhance external validity.

Practical Applications. From a practical perspective, coaches should integrate CS-specific drills and COD exercises into youth soccer conditioning. For U14 players, focus should be placed on foundational neuromuscular development, whereas U16 athletes can benefit from more specialized power and agility programs tailored to their physiological maturation stage. These adaptations can help bridge the gap between training content and match-specific demands, ultimately supporting better performance outcomes.

From an integrative perspective, it is essential to recognize that athletic performance and motor development cannot be evaluated solely through biomechanical or physiological parameters. The athlete’s biopsychosocial context directly influences neuroendocrine and immune function, thereby affecting both health and performance. Chronic stress, sleep quality, nutrition, emotional regulation, and the social environment act as contextual signals that shape brain plasticity, immune activity, and muscle recovery. Curved sprinting, which demands high levels of neuromuscular coordination and power, may be modulated by these systemic factors. This highlights the need for a holistic training approach that also considers emotional, behavioral, and environmental influences on young athletes. Such integration may not only optimize performance outcomes but also promote long-term, sustainable health.

## 5. Conclusions

This study provides new evidence on the multifactorial nature of curve sprint (CS) performance in youth male soccer players, focusing on two distinct age groups (U14 and U16). The results indicated that CS ability was associated with lower-limb explosive power, as measured by the countermovement jump (CMJ), and with agility and change-of-direction (COD) capacities, assessed through the IAT, 5-0-5, and V-Cut tests. These associations tended to be stronger in the U16 group, likely reflecting maturation-related improvements in neuromuscular coordination, eccentric control, and movement efficiency, as well as greater exposure to structured training. In contrast, VO_2_max showed no meaningful correlation with CS performance, suggesting that aerobic capacity may play a limited role in isolated, short-duration, high-intensity curvilinear sprint tasks that rely primarily on anaerobic power and neuromuscular efficiency. From a developmental perspective, these findings suggest that CS may serve as an indicator of age-related neuromuscular development and agility capacities. Practically, incorporating age-appropriate, curve sprint-specific training into youth conditioning programs may enhance multidirectional performance and movement efficiency. For younger players, training should emphasize general strength, coordination, and motor learning, while older athletes may benefit from targeted programs focusing on eccentric strength, agility, and technical efficiency. Overall, this study contributes to the growing understanding of the physical determinants of CS performance and supports the use of individualized, developmentally aligned training approaches to optimize performance in youth soccer. From a practical perspective, these findings may help coaches and conditioning specialists design age-appropriate, multidirectional training programs that enhance performance while managing asymmetries and neuromuscular load.

## Figures and Tables

**Table 1:** Comparison of Physical Performance Parameters Between U14 and U16 Soccer Players.

	U14	U16	*p*	Cohen’s d	Magnitude
VO_2_max (mL/kg/min) 95% CI:	44.93 ± 3.78 43.69–46.17	45.26 ± 3.43 44.14–46.39	0.687	−0.093 −0.542, 0.357	Small
CS GS (s) 95% CI:	5.66 ± 0.40 5.53–5.79	5.20 ± 0.56 5.02–5.39	**<0.001**	0.931 0.455, 1.402	Large
CS WS (s) 95% CI:	5.97 ± 0.42 5.83–6.11	5.42 ± 0.59 5.23–5.61	**<0.001**	1.076 0.591, 1.554	Large
CMJ (cm) 95% CI:	27.47 ± 4.99 25.84–29.11	33.58 ± 5.90 31.64–35.52	**<0.001**	−1.117 −1.598, −0.630	Large
IAT (s) 95% CI:	19.47 ± 1.59 18.95–19.99	18.44 ± 1.36 18.00–18.89	**<0.003**	0.695 0.229, 1.156	Medium
5-0-5 (s) 95% CI:	5.67 ± 0.54 5.50–5.85	5.33 ± 0.39 5.20–5.46	**<0.002**	0.736 0.268, 1.198	Medium
V-Cut (s) 95% CI:	8.09 ± 0.41 7.95–8.22	7.89 ± 0.54 7.72–8.07	0.081	0.406 −0.050, 0.859	Small

Note: *p* < 0.05 was considered statistically significant. GS: Good Side; WS: Weak Side; CMJ: Countermovement Jump; IAT: Illinois Agility Test; COD: Change of Direction; VO_2_max: Maximal Oxygen Uptake. Effect size interpretation: small (d = 0.2–0.49), medium (d = 0.5–0.79), large (d ≥ 0.8). Bold values indicate statistically significant differences (*p* < 0.05).

**Table 2:** Correlation Between Curve Sprint Times and Aerobic Power, Jump Performance, and Agility Tests in U14 Soccer Players.

U14		VO_2_max	CMJ	IAT	5-0-5	V-Cut
Curve Sprint GS	r CI	−0.297 −0.563, 0.025	−0.445 −0.670, −0.146	0.719 0.518, 0.844	0.438 0.137, 0.665	0.301 −0.020, 0.566
	*p*	0.070	<0.001 *	<0.001 *	0.006 *	0.066
Curve Sprint WS	r CI	−0.191 −0.482, 0.137	−0.399 −0.637,−0.091	0.669 0.445, 0.815	0.311 −0.010, 0.573	0.240 −0.086, 0.520
	*p*	0.250	0.013 *	<0.001 *	0.058	0.146

* FDR-adjusted q < 0.05 (Benjamini–Hochberg, within the correlation family). Bold values indicate statistically significant differences (*p* < 0.05).

**Table 3:** Correlation Between Curve Sprint Times and Aerobic Power, Jump Performance, and Agility Tests in U16 Soccer Players.

U16		VO_2_max	CMJ	IAT	5-0-5	V-Cut
Curve Sprint GS	r CI	−0.166 −0.461, 0.162	−0.661 −0.809, −0.432	0.785 0.621, 0.883	0.430 0.128, 0.659	0.477 0.186, 0.691
	*p*	0.319	<0.001 *	<0.001 *	0.007 *	0.002 *
Curve Sprint WS	r CI	−0.156 −0.453, 0.173	−0.729 −0.851, −0.534	0.775 0.606, 0.878	0.442 0.143, 0.668	0.478 0.187, 0.692
	*p*	0.351	<0.001 *	<0.001 *	0.005 *	0.002 *

* FDR-adjusted q < 0.05 (Benjamini–Hochberg, within the correlation family).

**Table 4:** Curve Sprint Directional Asymmetry (%).

Group	N	%DA	t	*p*	Cohen’s d
U14	38	5.70 ± 2.93	0.885	0.379	0.203 CI: [−0.248, 0.654]
U16	38	5.06 ± 3.37			

Note: *p* < 0.05 was considered statistically significant.

**Table 5:** Mixed-effects regression predicting curve-sprint time from neuromuscular and agility measures in U14 and U16 players.

Predictor (Unit)	Group	β (95% CI)	*p* (β)	Interaction	Practical (ΔX→ΔCS)	R^2^
CMJ (cm)	U14	−0.035 [−0.060, −0.010]	0.006	0.048	+5 cm → −0.174 s	0.522
	U16	−0.068 [−0.089, −0.047]	<0.001		+5 cm → −0.338 s	0.522
Illinois (s)	U14	+0.179 [+0.115, +0.244]	<0.001	0.003	+1.0 s → +0.179 s	0.662
	U16	+0.329 [+0.254, +0.405]	<0.001		+1.0 s → +0.330 s	0.662
COD505 (s)	U14	+0.286 [+0.016, +0.556]	0.038	0.128	+1.0 s → +0.286 s	0.367
	U16	+0.643 [+0.271, +1.015]	<0.001		+1.0 s → +0.643 s	0.367
VCut (s)	U14	+0.269 [−0.081, +0.620]	0.132	0.292	+1.0 s → +0.269 s	0.369
	U16	+0.506 [+0.240, +0.772]	<0.001		+1.0 s → +0.506 s	0.369
VO_2max_ (mL × kg^−1^ × min^−1^)	U14	−0.026 [−0.068, +0.015]	0.211	0.988	+5 mL·kg^−1^·min^−1^ → −0.132 s	0.267
	U16	−0.027 [−0.072, +0.018]	0.248		+5 mL·kg^−1^·min^−1^ → −0.134 s	0.267

R^2^ = marginal (fixed effects); random intercept for player; Side (Weak vs. Good) covariate included. Model: CS (s) ~ Predictor + Age group (U16 = 1) + Side (Weak = 1) + Predictor × Age + (1|Player); REML estimation; Satterthwaite df; two-tailed tests; 95% CIs.

## Data Availability

The original contributions presented in this study are included in the article. Further inquiries can be directed to the corresponding authors.
